# Correction: Iqbal et al. Floating ZnO QDs-Modified TiO_2_/LLDPE Hybrid Polymer Film for the Effective Photodegradation of Tetracycline under Fluorescent Light Irradiation: Synthesis and Characterisation. *Molecules* 2021, *26*, 2509

**DOI:** 10.3390/molecules30071462

**Published:** 2025-03-26

**Authors:** Anwar Iqbal, Usman Saidu, Farook Adam, Srimala Sreekantan, Noorfatimah Yahaya, Mohammad Norazmi Ahmad, Rajabathar Jothi Ramalingam, Lee D. Wilson

**Affiliations:** 1 School of Chemical Sciences, Universiti Sains Malaysia, Gelugor, Penang 11800, Malaysia; usmaniyya2000@gmail.com (U.S.); farook@usm.my (F.A.); 2 School of Materials & Mineral Resources Engineering, Universiti Sains Malaysia, Nibong Tebal, Penang 14300, Malaysia; srimala@usm.my; 3 Integrative Medicine Cluster, Advanced Medical and Dental Institute, Universiti Sains Malaysia, Bertam, Kepala Batas, Penang 13200, Malaysia; noorfatimah@usm.my; 4 Experimental and Theoretical Research Lab, Department of Chemistry, Kulliyyah of Science, Kuantan, Pahang 25200, Malaysia; mnorazmi@iium.edu.my; 5 Surfactant Research Chair, Chemistry Department, College of Science, King Saud University, P.O. Box 2455, Riyadh 11451, Saudi Arabia; 6 Department of Chemistry, University of Saskatchewan, 110 Science Place, Room 165 Thorvaldson Building, Saskatoon, SK S7N 5C9, Canada

**Error in Figures**

In the original publication [1], Figure 1 had a low image resolution. Below is an improved version of Figure 1 with a better resolution.

Furthermore, there were mistakes in Figures 10 and S4 as published. The figures have been removed. With this correction, the order of some figures has been adjusted accordingly. 

**Text Correction**

There was an error in the original publication [1]. The discussion on LC/TOF/MS analysis was noticed to have flaws that were found in the last sentence in the Abstract, the last sentence in Section 1 Introduction, the last paragraph in Section 2.2.5, the last paragraph in Section 3.6, and the last sentence in Supplementary Materials. The above parts which were related to the LC/TOF/MS analysis have been removed. 

**References**

With this correction, the order of some references has been adjusted accordingly. The authors state that the scientific conclusions are unaffected. This correction was approved by the Academic Editor. The original publication has also been updated.

## Figures and Tables

**Figure 1:**
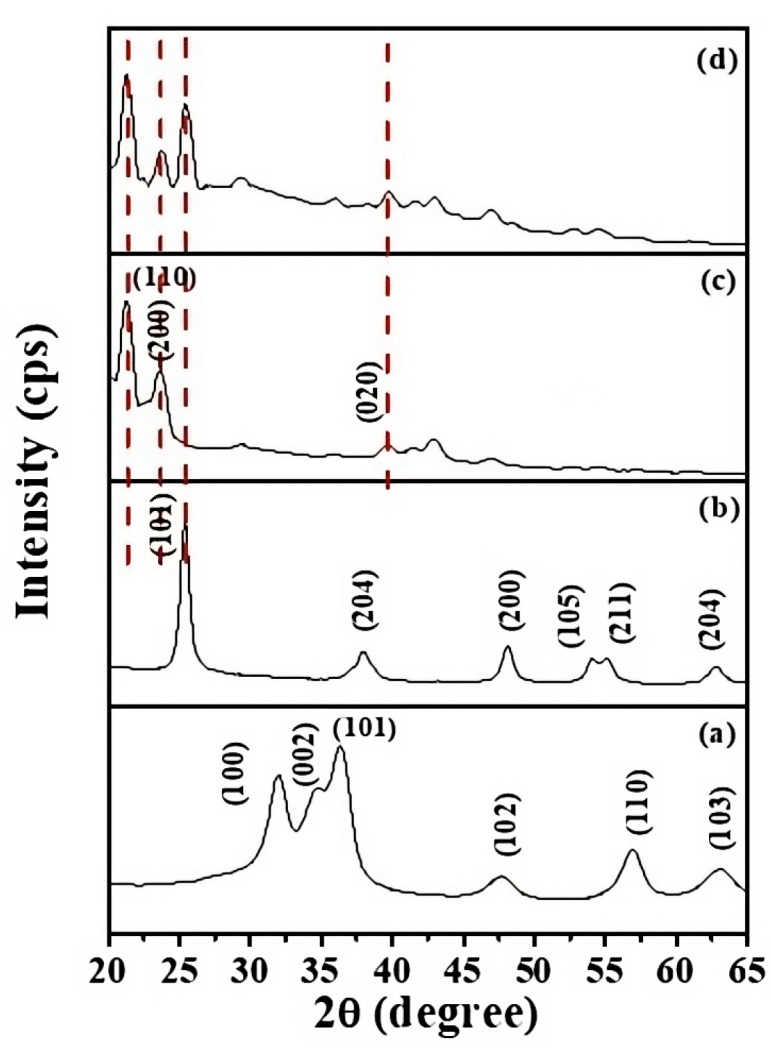
XRD diffractogram of materials: (**a**) ZnO QDs, (**b**) ZT, (**c**) bare LLDPE, and (**d**) 8%-ZT@LLDPE.
