# Supplementary material for: Floating ZnO QDs-Modified TiO2/LLDPE Hybrid Polymer Film for the Effective Photodegradation of Tetracycline under Fluorescent Light Irradiation: Synthesis and Characterisation
Source: Molecules. 2021 Apr 25;26(9):2509. doi: 10.3390/molecules26092509 (PMC8123277; doi:10.3390/molecules26092509)
Supplement: Supplementary file 1 [file molecules-26-02509-s001.zip › molecules-1165168-supplementary.pdf]

Floating ZnO QDs-modified TiO_2/ LLDPE hybrid polymer film for the effective photodegradation of tetracycline under fluorescent light irradiation: Synthesis and Characterization

Anwar Iqbal[a*], Usman Saidu[a], Srimala Sreekantan[b], Noorfatimah Yahaya[c], Mohammad Norazmi Ahmad[d], R. Jothi Ramalingam[e*], and Lee D. Wilson[f*]

[1] School of Chemical Sciences, Universiti Sains Malaysia, Penang 11800, Malaysia, usmaniyya2000@gmail.com (U.S.); farook@usm.my (F.A.)
[2] School of Materials & Mineral Resources Engineering, Universiti Sains Malaysia, Engineering Campus, 14300 Nibong Tebal, Seberang Perai Selatan, Pulau Pinang, Malaysia, srimala@usm.my
[3] Integrative Medicine Cluster, Advanced Medical and Dental Institute, Universiti Sains Malaysia, 13200, Kepala Batas, Pulau Pinang, Malaysia, noorfatimah@usm.my
[4] Experimental and Theoretical Research Lab, Department of Chemistry, Kulliyyah of Science, 25200 Kuantan, Pahang, Malaysia, mnorazmi@iium.edu.my
[5] Surfactant Research Chair, Chemistry Department, College of Science, King Saud University, P.O. Box. 2455, Riyadh 11451, Kingdom of Saudi Arabia
[6] Department of Chemistry, University of Saskatchewan, 110 Science Place, Room 165 Thorvaldson Building, Saskatoon, SK. Canada S7N 5C9
* Correspondence: anwariqbal@usm.my (A.I.) Tel. +604-653 3565; jrajabathar@ksu.edu.sa (R.J.R.); lee.wilson@usask.ca (L.D.W.) Tel. +1-306-966-2961

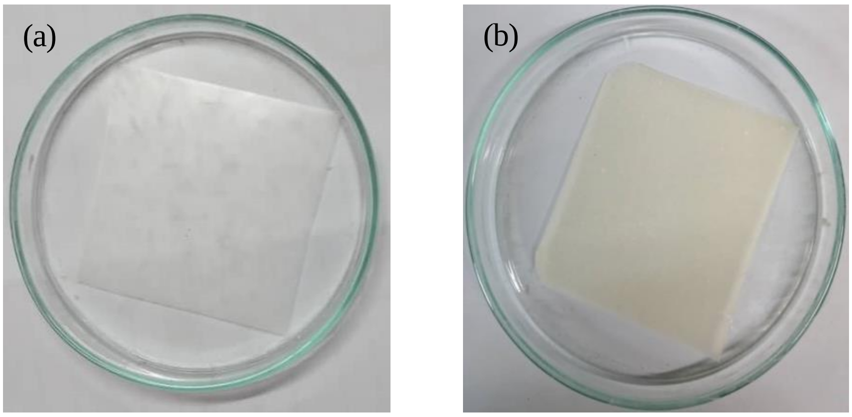

Figure S1: Photographs of (a) bare LLDPE and (b) 8%-ZT@LLDPE hybrid films.

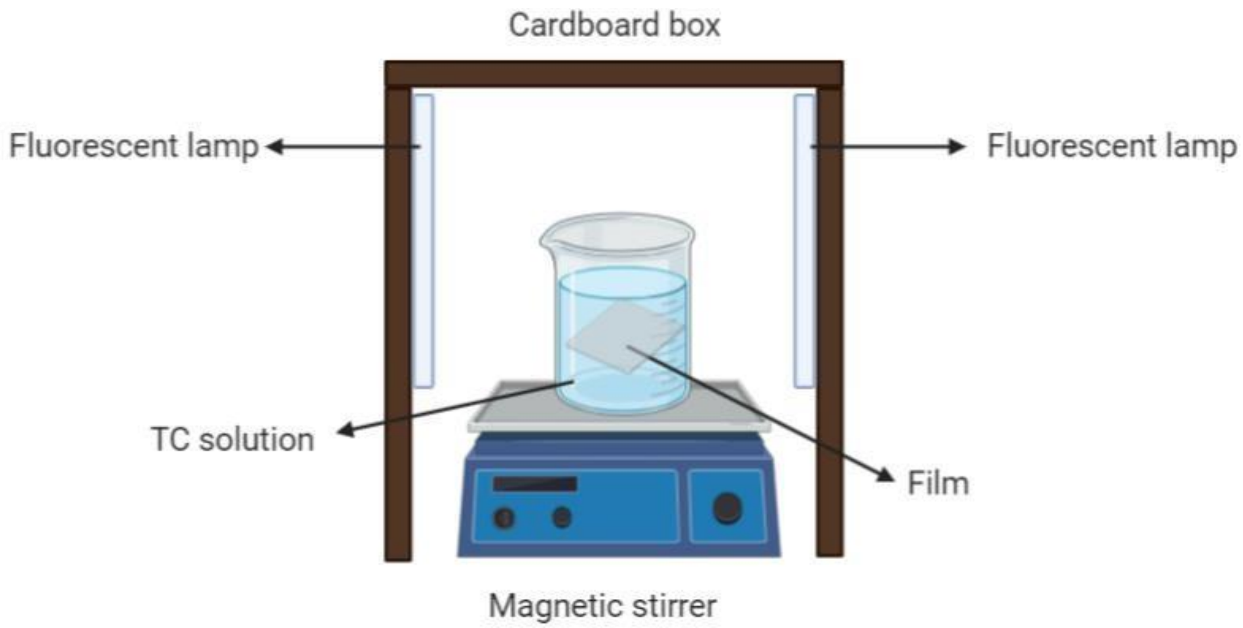

Figure S2: Schematic diagram of the photocatalytic reactor for the degradation of TC using the hybrid LLDPE film.

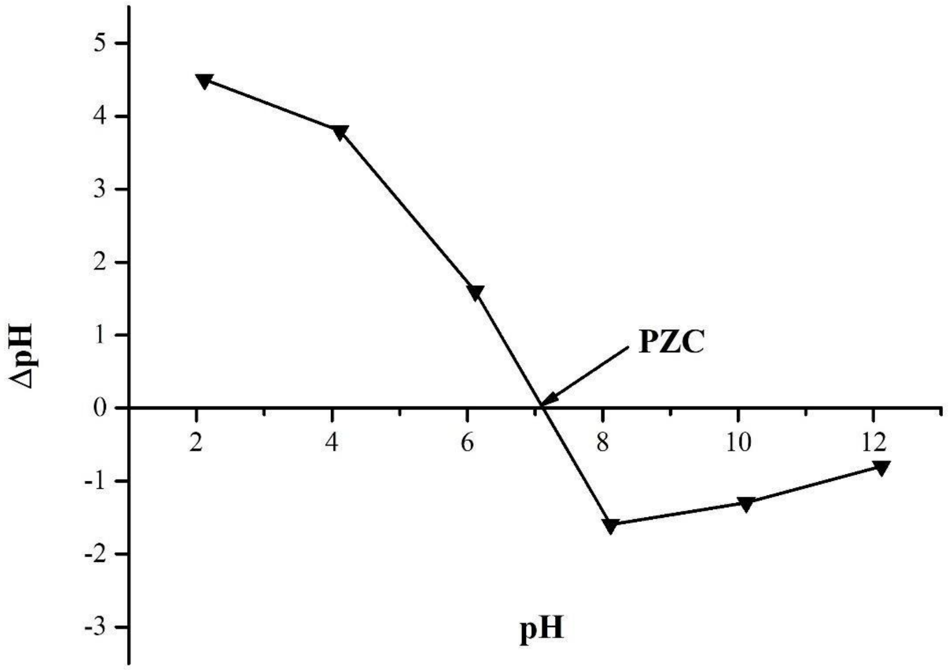

Figure S3: The pH point of zero charge (pH_{PZC}) of ZT nanocomposites photocatalyst.
